# Adenocarcinoma arising in a heterotopic pancreas (Heinrich type III): a case report

**DOI:** 10.1186/1752-1947-4-39

**Published:** 2010-02-06

**Authors:** Yoshihiro Inoue, Michihiro Hayashi, Yoshifumi Arisaka, Kazuhide Higuchi, Yutaro Egashira, Nobuhiko Tanigawa

**Affiliations:** 1 Department of General and Gastroenterological Surgery, Osaka Medical College Hospital, 2-7 Daigaku-machi, Takatsuki City, Osaka 569-8686, Japan; 2 Department of Internal Medicine, Osaka Medical College Hospital, 2-7 Daigaku-machi, Takatsuki City, Osaka 569-8686, Japan; 3 Department of Pathology, Osaka Medical College Hospital, 2-7 Daigaku-machi, Takatsuki City, Osaka 569-8686, Japan

## Abstract

**Introduction:**

Heterotopic pancreatic cancer in the duodenum is a very rare disease. Only twelve cases have been reported worldwide to date. We report a rare case of malignant transformation of heterotopic pancreas (Heinrich type III) in the duodenum with long-term survival of the patient, and review the 12 cases in the literature.

**Case presentation:**

A 75-year-old Japanese man was admitted to our hospital complaining of nausea and vomiting. Endoscopy and upper gastrointestinal contrast study showed marked duodenal stenosis. A pylorus-preserving pancreaticoduodenectomy was performed. Histopathological examination of the surgically resected specimen showed malignant transformation of heterotopic pancreas (Heinrich type III) in the duodenum. The postoperative course was uneventful, and the patient was discharged from the hospital on postoperative day 30. He is well and shows no signs of recurrence at the time of writing, six years after the surgery.

**Conclusion:**

Adenocarcinoma arising within the heterotopic pancreas appears to be rare. It is difficult to obtain a correct diagnosis preoperatively. The management of heterotopic pancreas depends on the presence or absence of symptoms. If the patient is asymptomatic or benign, conservative treatment with regular follow-up is recommended. When the patient is symptomatic or there is a suspicion of malignancy, surgical management with intra-operative frozen section diagnosis is indicated.

## Introduction

Shultz was first to describe heterotopic pancreas in 1727, although histological data was not available until the report by Klob in 1859. Heterotopic pancreas is also called aberrant or ectopic pancreas. Although heterotopic pancreas is rarely found during surgical exploration of the upper part of the abdomen, occurring in less than 0.5% of abdominal laparotomies, heterotopic pancreas is found in 1-2% of autopsies [1]. It is usually asymptomatic, but most diseases affecting the pancreas can occur within these heterotopias. Heterotopic pancreas has been classified by Heinrich [2]. Adenocarcinoma arising within heterotopic pancreas appears to be a rare occurrence, with fewer than 30 cases being reported in the literature worldwide.

## Case presentation

**Figure 1:**
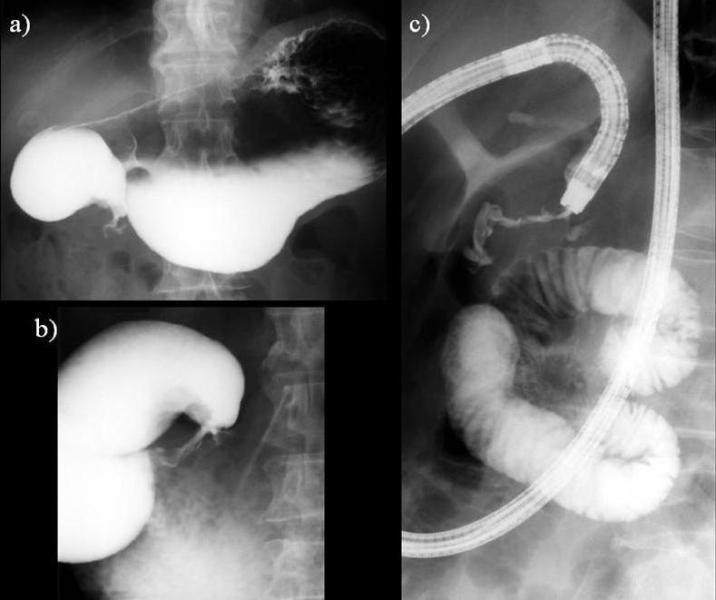
**(a, b, c)**. Upper gastro-intestinal contrast study revealed smooth stenosis of the second part of the duodenum **(a)**, with a dilatation of the duodenal bulb **(b) **due to elevated lesion of 3 cm in diameter **(c, arrow)**, and passage of the contrast medium to anal side of the stenosis was markedly disturbed.

**Figure 2:**
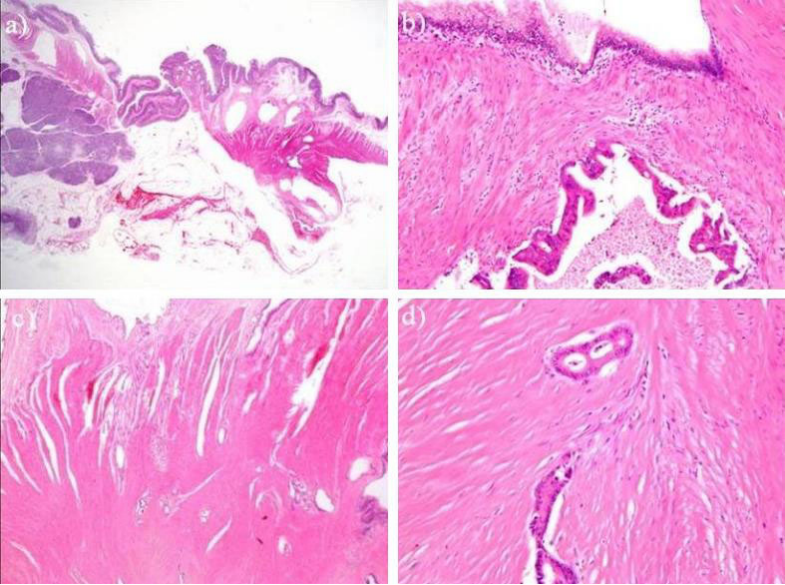
**(a, b, c, d)**. Pathological findings of the resected specimen showed numerous cystically dilated ducts surrounded by numerous proliferating smooth-muscle bundles without islets or acini from the submucosal layer to the proper muscular layer **(a, b)**. Well differentiated adenocarcinoma cells proliferating diffusely in an area ranging from the submucosal layer to the subserosa was observed in a direct transition to dysplastic ductal structures **(a, c, d)**. (hematoxylin-eosin, a: × 20, b: × 100, c: × 100, d: × 200)

A 75-year-old Japanese man was admitted to our hospital, complaining of nausea and vomiting. His previous personal and family history was noncontributory. Findings of blood examination were almost normal, including carcinoembryonic antigen and carbohydrate antigen 19-9. Physical examination revealed cool moist skin, pulse rate of 84 per minute and blood pressure of 131/76 mmHg. Endoscopy showed marked duodenal obstruction, though the mucosa of the stenotic site seemed almost intact. The biopsy specimen obtained from stenotic site revealed regenerative mucosa without malignancy. Upper gastrointestinal contrast study showed smooth stenosis of the second part of the duodenum with a dilatation of the duodenal bulb due to elevated lesion of 3 cm in diameter (Figure. 1). Abdominal computed tomography (CT) showed a homogeneously enhanced mass of 2 cm in diameter with clear margin within the wall of the thickened duodenum. The tumor was low (poorly stained) on arterial phase and well enhanced on the equilibrium phase by contrast medium. Magnetic resonance imaging (MRI) revealed low-intensity mass on T1-shifted phase and high-intensity on T2-shifted phase. No abnormal findings were shown in other abdominal organs. Under the diagnosis of duodenal carcinoma arising from descending portion of the duodenum, malignant lymphoma or gastrointestinal stromal tumor of the duodenum, pylorus-preserving pancreaticoduodenectomy was performed. On postoperative histological examination, heterotopic pancreatic tissue was detected just from the submucosal layer to the proper muscular layer, which was consisted of numerous ducts without islets and acini, and thus diagnosed as Heinrich type III heterotopic pancreas. A transition to dysplastic ductal structures was well-differentiated adenocarcinoma cells proliferating diffusely in areas ranging from the submucosal layer to the subserosa, resulting in a diagnosis of adenocarcinoma arising from heterotopic pancreas in the duodenum (Figure. 2). On microscopic examination, the involvement of lymph nodes No.13 was confirmed. The postoperative course of this patient was uneventful and he was discharged from the hospital on postoperative day 30.

## Discussion

Heterotopic pancreas with its complete absence of both anatomic and vascular connections to the main pancreas is considered to result from the altered development of one of the two primitive pancreatic anlagens that normally fuse to form the uncinate-head and body-tail of the normal gland. This alteration of embryonic growth results in an ectopic rest being dropped from the dorsal pancreatic rudiment. It is then left to develop in a site away from the usual location of the body and tail of the pancreas, therefore, heterotopic pancreas arose in surrounding or adjacent organs.

Heterotopic pancreas has been reported by Busarb *et al. *[3] to occur at a large number of sites within the abdomen, involving not only the hollow viscera but also the omentum, mesentry, and spleen. Most cases reported in the literature occurred in duodenum (29.3%), stomach (27.4%), jejunum (15.7%), ileum (5.9%), Meckel's diverticulum (5.1%), and gallbladder (2.7%). The majority of reports refer to the stomach, because the chance of endoscopic observation and organ resection is much more frequent than in other organs. Heterotopic pancreas is mainly single, though multiple in some cases, with the size of the nodule ranging from 0.1 cm to 2.0 cm but usually smaller than 1 cm [4]. The classic radiologic and endoscopic features of heterotopic pancreas are awell-circumscribed intramural nodule, locating mainly in the submucosal layer, with a central umbilication that may be the site of ductal drainage to the mucosal surface.

The majority of cases appear to be asymptomatic, and most symptoms attributable to heterotopic pancreas include non-specific ones, such as epigastric pain, nausea, and vomiting. Heterotopic pancreas may undergo acute and chronic pancreatitis, bleeding and perforation, gastric outlet obstruction, pseudocyst formation, intussusceptions and, rarely, malignant transformation.

Heterotopic pancreas has been classified into three types, according to its constitutional components by Heinrich [2]. Namely, Type I consists of typical pancreatic tissues with acini, ducts, and islet cells similar to those seen in normal pancreas. Type II is composed of pancreatic tissues with ducts and acini. Islet cells are absent. Type III is composed of pancreatic tissue with large numbers of ducts and few acini. Class III is characterized by an absence of islet cells, rare acini, and numerous ducts, some of which are cystically dilated. Some Class III cases lack acinar tissue entirely. Furthermore, heterotopic pancreas is similar to so-called adenomyoma. Adenomyoma, first described histologically by Maugnus-Alsleben [5], is characterized by cystic dilation and poorly differentiated ducts surrounded by proliferating numerous smooth-muscle bundles without acini or islets in the submucosal layer. Heinrich Class III is virtually almost the same as adenomyoma in this regard, and thus adenomyoma can be considered a subtype of heterotopic pancreas [6].

A literature search of Medline, from 1963 to 2007, with the key words "heterotopic", "aberrant", "ectopic", "duodenum", "pancreas", and "cancer" yielded twelve reports of malignant transformation of heterotopic pancreas in the duodenum (Table. 1) [7-9]. It is not always easy to make a histological determination that a carcinoma has arisen from a heterotopic pancreatic tissue, neither invaded from neighboring organs nor metastasized from other organs. Guillou *et al. *[10] described that the possibility of heterotopic pancreatic tissue origin is acceptable only if following three conditions are met: [1], the tumor must be found within or close to the heterotopic pancreatic tissue; [2], direct transition between pancreatic structures and the carcinoma must be observed; and [3], the non-neoplastic pancreatic tissue must comprise at least fully developed acini and ductal structures. Our case satisfied these three conditions.

**Table 1:** Review of cases of carcinoma of duodenal heterotopic pancreas

Case	Age/Sex	Clinical symptoms	Classification	Author (year)
1	28/F	epigastric pain	II	Bookman MR (1932)

2	55/M	vomiting	III	Potet F (1970)

3	54/M	vomiting	III	Leger L (1976)

4	72/M	pain	unknown	Ikeda R (1980)

5	56/M	jaundice	II	Al Jitawi SA (1984)

6	76/M	right hypochondralgia	unknown	Wakahara T (1988)

7	26/F	epigastric pain	unknown	Franke U (1989)

8	68/M	body weight loss	I	Senmaru N (1995)

9	81/M	appetite loss	I	Waku T (1996)

10	64/M	unknown	unknown	Nam JY (2003)

11	75/M	melena	III	Kaneko K (2006)

12	72/M	pain and jaundice	unknown	Tison C (2007)

Our case was discovered not at an earlier stage, but as an advanced cancer that simulated a T3 pancreatic cancer during operation. Mainly, it is because the tumor existed in an area ranging from the submucosal layer to the proper muscular layer, proliferating invasively from the proper muscular layer to the serosa without any clinical symptoms for a long time. We performed pancreaticoduodenectomy with thorough dissection of the regional lymph nodes, and considered the possibility of it having a malignant character though without intraoperative frozen section diagnosis. Although prognosis of malignant heterotopic pancreas is poor in general, our case is alive and well with no sign of recurrence six years after the surgery.

## Conclusion

The management of heterotopic pancreas depends on the presence of symptoms. If the patient is asymptomatic or benign, conservative treatment with regular follow-up is recommended. When the patient is symptomatic or the heterotopic is suspected of being malignant, surgical management with intraoperative frozen section diagnosis is needed.

## Consent

Written consent was obtained from the patient for publication of the case report and any accompanying images. A copy of the written consent is available for review by the Editor-in-Chief of the journal.

## Competing interests

The authors declare that they have no competing interests.

## Authors' contributions

YI conceived the study concept and design, was involved with patient care and drafted the manuscript and literature review. MH, YA, KH, and YE were involved with formation of the study concept and design, patient care and drafting of the manuscript and the literature review. NT carried out the operation on the patient and was the main contributor in the writing of the manuscript. All authors have read and approved the final version of the manuscript.
